# Synthetic Efforts for Stereo Structure Determination of Cytotoxic Marine Natural Product Pericosines as Metabolites of *Periconia* sp. from Sea Hare

**DOI:** 10.3390/ijms9030401

**Published:** 2008-03-24

**Authors:** Yoshihide Usami, Hayato Ichikawa, Masao Arimoto

**Affiliations:** Osaka University of Pharmaceutical Sciences, 4-20-1 Nasahara, Takatsuki, Osaka 569–1094, Japan

**Keywords:** marine natural product, antitumor, pericosine, structure determination, total synthesis, carbasugar

## Abstract

Pericosines are unique C_7_ cyclohexenoid metabolites of *Periconia byssoides* OUPS-N133 fungus that was originally isolated from the sea hare, *Aplysia kurodai*. Pericosines show significant *in vitro* cytotoxicity against P388 lymphocytic leukemia cells. Pericosine A, in particular, shows the most potent activity and significant *in vivo* antitumor activity against P388 cells. Thus, pericosines are promising candidates for seed compounds of anticancer drugs. However, before the total syntheses of pericosines were accomplished, their stereo structures could not be determined by spectral analyses because they have multi-functionalized cyclohexenoid structures with torsional strain. In this review, synthetic efforts for pericosines in this decade are surveyed.

## 1. Introduction

Synthetic studies of carbasugars have been progressing on a worldwide scale [1–3]. Carbasugars are a class of carbocyclic analogues of monosaccharides in which oxygen atom in the ring is replaced with a carbon atom. Because of this, they are also called pseudo-sugars. Carbasugars exhibit gycosidase inhibitory, antitumor (including anticancer), antiviral, antifungal, antibacterial, and antimalarial activities. Therefore, the synthetic study of carbasugars is extremely important for the discovery of new drugs, including cancer preventive drugs. Mammals lack the shikimate pathway found in plants, fungi, microorganisms, and apicomplexan parasites. The chances of drug discovery are improved when shikimate analogues are focused on.

**Figure 1.**
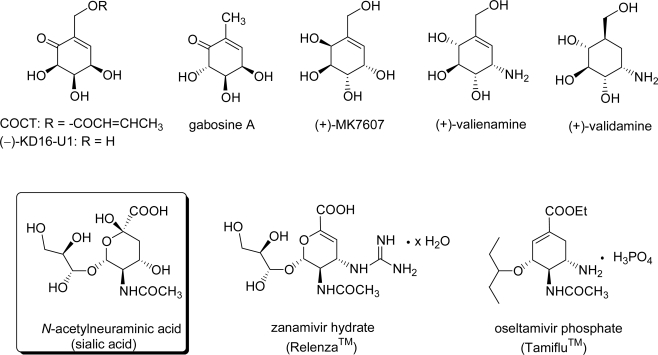
Structures of bioactive carbasugars as synthetic targets.

Figure 1 illustrates the structures of representative carbasugars as synthetic targets, including 2-crotonyloxy-(4R,5R,6R)-4,5,6-trihydroxy-cyclohex-2-enone (COCT) [4–15], [(−)-KD16-U1 [gabosine A [16–21], (+)-MK7607 [22, 23], (+)-valienamine [24–29], (+)-validamine [29–31], Relenza® [32], and Tamiflu® [33–40], COCT [41], (−)-KD16-U1 [42], and gabosines [43], which were isolated from cultures of *Streptomyces spp*., are potential glyoxalase inhibitors and therefore show promise as anticancer agents. (+)-MK7607, which was isolated from cultures of *Curvularia eragrostidis* D2452, showed herbicidal activity [44]. (+)-Valienamine and (+)-validamine were obtained from the degradation of antifungal antibiotic validamycin A by *Pseudomonas denitrificans* [45–47]. Valienamine is also a component of ascarbose, a potent α-glucosidase inhibitor [48].

Relenza and Tamiflu are the most popular anti-influenza drugs today and are therefore attractive target molecules for synthetic studies. Relenza is a potent anti-influenza drug designed from the transition structure of *N*-acetylneuramic acid-neuramidase complex based on X-ray analysis [32]. The oral anti-influenza drug Tamiflu has received immense attention for its effect on avian flu. To this day, however, the synthesis of Tamiflu remains an enigma for synthetic organic chemists. The independent asymmetric total synthesis of Tamiflu by Corey [35] and Shibasaki [36, 37] from non-natural starting material, which was published in JACS, was a hot topic in 2006. In 2007, Fukuyama and co-workers reported the practical synthesis of Tamiflu [38].

**Figure 2.**
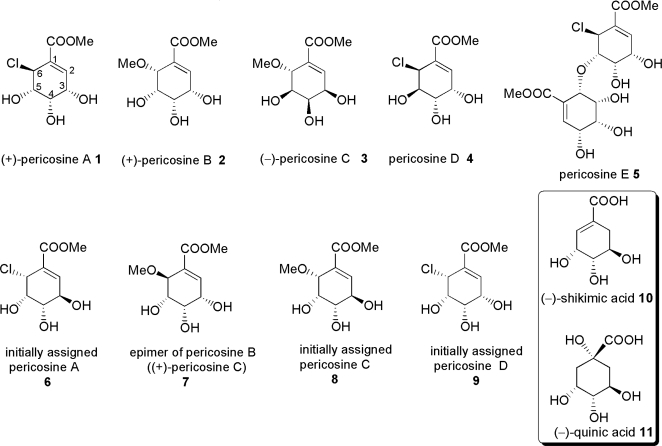
Structures of pericosines and analogues.

In the past several decades, scientists working on new drug discovery have extended their research field to include the ocean because of its vast biological/chemical diversity and the fact that it composes almost 70% of Earth’s surface. Marine natural products chemistry has produced a number of promising candidates for anticancer drugs and several of them have undergone pre- or phases I-III clinical trials [49, 50]. Recently, however, a new tide has emerged, which involves the study of metabolites of microorganisms from marine sources [51–53]. It must be emphasized that researchers involved in this endeavor have been attempting preservation of environmental habitat in the ocean. In 1997, Numata and co-workers isolated unique C_7_ cyclohexenoid-type metabolites from *Periconia byssoides* OUPS-N133 fungus that was originally isolated from the sea hare, *Aplysia kurodai*, and designated them as pericosines A **1** and B **2** (Figure 2), together with some macrospherides [54]. Full details of the isolation of pericosines A-E **1–5** were very recently reported by Numata and co-workers [55]. Pericosines showed significant *in vitro* cytotoxicity against P388 lymphocytic leukemia cells. Particularly, **1** was reported to have inhibitory activity against protein kinase and topoisomerase II in addition to significant *in vivo* antitumor activity against P388 cells. Thus, pericosines are thought to be promising candidates for seed compounds of anticancer drugs. However, total syntheses were required to confirm the stereo structures of most pericosines because the multi-functionalized cyclohexenoid structures with severe torsional strain have made structure determination by spectral analyses difficult. In this review, synthetic efforts to produce pericosines and analogues **1**, **2**, **4–7**, and **9** from (−)-shikimic acid **10** or (−)-quinic acid **11** are surveyed comprehensively, together with the discovery of natural compounds.

## 2. Isolation of Pericosines from *Periconia byssoides* [54, 55]

Numata and co-workers reported the isolation of C_7_ cyclohexenoids designated as pericosines A-E **1–5** in 1997 and 2007, as summarized below. The fungal strain *P. byssoides* OUPS-N133 was isolated from a culture of *A. kurodai* by bioactivity-guided cytotoxicity assay using P388 cells. The fungal strain was cultured in artificial seawater medium containing 1% malt extract, 1% glucose, and 0.05% peptone adjusted to pH 7.5 at 27°C. After 4 weeks, mycelia obtained by filtration of the broth were extracted with AcOEt. The AcOEt extract was separated by gel filtration through Sephadex LH-20 using MeOH-CH_2_Cl_2_ (1:1), followed by silica gel column chromatography with CH_2_Cl_2_-MeOH gradient solvent system. This separation was performed along with *in vitro* P388 cytotoxicity assay. The active fraction that was eluted with 10% MeOH-CH_2_Cl_2_ was further urified by reverse phase preparative HPLC using the MeOH-H_2_O solvent system to afford **1–5**. Spectroscopic analyses including NMR, MS, and IR led to the elucidation of their structures. Early studies, however, indicated the structure of pericosine A as **6**, that of pericosine C as **8**, and that of pericosine D as **9** [56, 57]. It is difficult to determine the relative configuration of such compounds as pericosines because they possess a multi-functionalized cyclohexenoid core with torsional strain. In their most recent report, the authors stated that they elucidated the structures of pericosines, except for pericosine A, by spectroscopic analyses. They determined the relative chemistry of pericosine B **2** on the basis of NOE between H-3 and H-5 observed in its acetonide **2a** and small coupling constants *J*_3–4_ = 5.7 Hz, *J*_4–5_ = 3.1 Hz, and *J*_5–6_ = 4.1 Hz. Long-range coupling between H-4/H-6, H-2/H-6, and H6/H-2 observed in **2a** also supported their proposed configuration. Similar NOE between H-3 and H-5 and long-range coupling between H-2/H-6 and H-4/H-6 were observed in the spectra of **2**, suggesting similar configuration. Pericosine C **3**, which has the same planar structure as pericosine B **2**, was transformed into acetonides **3a** and **3b**. Spectral analysis indicated that the 3,4,5-trihydroxyl groups on the cyclohexene ring had *cis, cis* configuration, and the stereochemistry at C-6 was determined to be different from that of **2**. Finally, its identity was confirmed by comparing with synthesized compound **7**. Interestingly, pericosine C exists as an enantiomeric mixture of **3** and **7**, based on comparison of specific rotation values between natural pericosine C ([α]_D_^25^−4.8) and synthetic **7** ([α]_D_^25^+35.1). The relative chemistry of pericosine D **4** was determined on the basis of NOE between one acetonide-methyl/H-3, H-4 and another acetonide-methyl/H-5 observed in the NOESY spectrum of its 3,4-acetonide. The relative chemistry of pericosine E **5** was confirmed by X-ray analysis. Pericosine E **5** also exists as an enantiomeric mixture.

Pericosines are responsible for the cytotoxicity of the extract of *P. byssoides*. The ED_50_ values of pericosines are presented in Table 1. Compounds **1**, **2**, and **4** exhibited significant *in vitro* cytotoxicity against P388 cells. Most potent **1** also showed *in vivo* antitumor activity against P388 cells. Furthermore, **1** inhibited protein kinase EGFR and human topoisomerase II and showed selective growth inhibition against human cancer cell lines HBC-5 and SNB-75. Thus, pericosines were proved to be promising candidates for seed compounds of cancer preventive drugs.

## 3. Synthetic Efforts for Pericosines

As described above, the syntheses of pericosines are required not only to determine absolute configuration but also to confirm relative stereochemistry. The following are synthetic efforts by several groups carried out so far.

### 3.1. Total synthesis of pericosine B [58]

#### 3.1.1. Donohoe’s approach

**Scheme 1.**
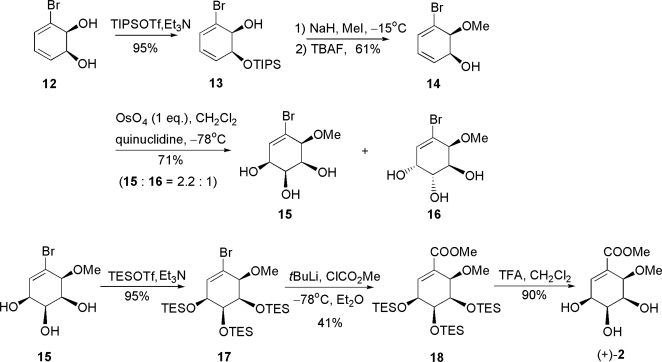
Successful total synthesis of (+)-pericosine B by Donohoe and co-workers.

Donohoe and co-workers were the first to report the total synthesis of pericosine B (+)-**2** in 1998. This is the only successful synthesis to date and has been achieved by using their original hydrogen-bond-directed dihydroxylation with osmium tetroxide to give *cis, cis*-triol. The synthesis is summarized in Scheme 1.

Commercially available (+)-dihydroxybromocyclohexadiene **12** was selectively protected to give silyl ether **13**, which was then *O*-methylated with NaH and MeI and deprotected with TBAF to give alcohol **14**. The subsequent hydrogen-bond-directed dihydroxylation of **14**, which was the key step of this total synthesis, was carried out with stoichiometric osmium tetroxide and one equivalent of quinuclidine to give syn, syn- and syn, anti-triols, **15** and **16**, in 2.2: 1 ratio. Separated **15** was protected with triethylsilyl (TES) groups to give **17**, and **17** was methoxycarbonylated to yield **18**. The synthesis was terminated by the addition of TFA to afford **2**, which had the same spectroscopic data including specific rotation as those of the natural product, leading to the conclusion that the absolute stereochemistry is methyl (3*S*,4*S*,5*S*,6*R*)-3,4,5-trihydroxy-6-methoxycyclohexene-1-carboxylate. The problems of this synthesis included poor stereoselectivity in the crucial step and use of a stoichiometric amount of highly toxic osmium tetroxide.

#### 3.1.2. Okamura’s approach [59]

**Scheme 2.**
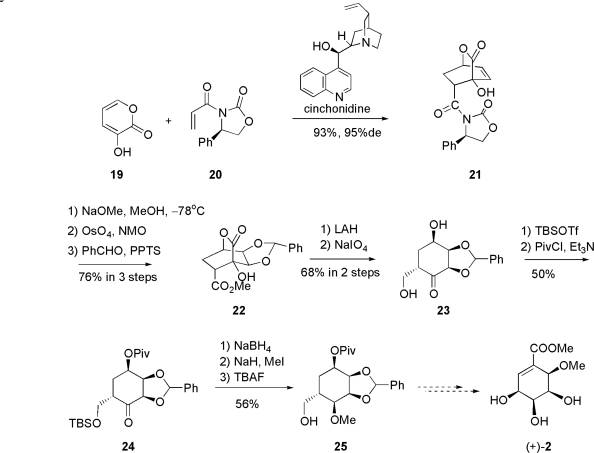
Okamura’s approach.

Okamura and co-workers attempted the total synthesis of pericosine B also in 1998. The key reaction of their synthesis was the asymmetric Diels-Alder reaction of 3-hydroxy-2-pyrone **19** with a basic catalyst. Their synthetic approach is illustrated in Scheme 2.

The enantioselective Diels-Alder reaction between 3-hydroxy-2-pyrone **19** and acrylate with chiral auxiliary **20** was carried out in the presence of cinchonidine as basic catalyst to give endo-adduct **21** in 93% yield with 95% de. Adduct **21** was then treated with NaOMe to remove chiral auxiliary and the resultant methyl ester was dihydroxylated with catalytic osmium tetorxide and NMO to afford exo-diol, which was protected as benzylidene acetal **22**. Reduction with LiAlH_4_ followed by treatment with NaIO4 gave diol **23**, which was successively protected with a primary hydroxyl group such as TBS-ether followed by a secondary hydroxyl group such as pivaloyl ether to give ketone **24**. Stereoselective reduction with NaBH_4_ afforded β-hydroxyalcohol, which was then *O*-methylated and deprotected with TBAF to give hydroxymethylether **25**. Unfortunately, the completion of this synthesis from the intermediate has not been reported so far.

#### 3.1.3. Usami’s approach [60]

**Scheme 3.**
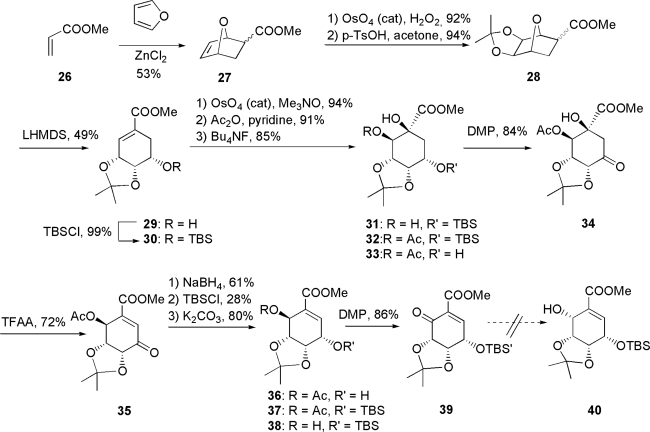
Usami’s approach.

An approach taken by our group toward pericosine B is shown in Scheme 3. Racemic Diels-Alder adduct **27**, which was formed by reacting methyl acrylate **26** with furan in the presence of ZnCl_2_ as Lewis acid catalyst, was dihydroxylated with H_2_O_2_ and catalytic osmium tetroxide with excellent *exo*-face selectivity. Acetonides **28** derived from the mixture of diastereoisomers **27** were treated with LHMDS in THF at −78°C to give methyl 5-epishikimate derivative **29** as a single product, whose hydroxyl group at C-5 was protected as silyl ether **30**. Compound **30** was dihydroxylated with osumium tetroxide to give sole product **31** with the desired stereochemistry. After many examinations of regioselective *O*-methylation and *O*-acylation of **31**, it was protected at C-2 by acetylation to give **32**, then deprotected with 5-TBS ether to give alcohol **33**, which in turn was oxidized with Dess-Martin periodinane to give β-hydroxyketone **34**. α,β-Unsaturated ketone **35** obtained by adding TFAA to **34** was reduced with NaBH_4_ to give alcohol **36** with the desired stereochemistry of **37**, followed by protection and deprotection. Alcohol **38** was oxidized with Dess-Martin periodinane to give unstable α,β-unsaturated enone **39**, which could not be purified by silica gel chromatography. Crude **39** was reduced to generate hydroxyl group with the desired stereochemistry. However, not our objective reaction but an unexpected reaction occurred to yield **40**. This work was published in 2004.

#### 3.1.4. Garcia Ruano’s approach [61]

**Scheme 4.**
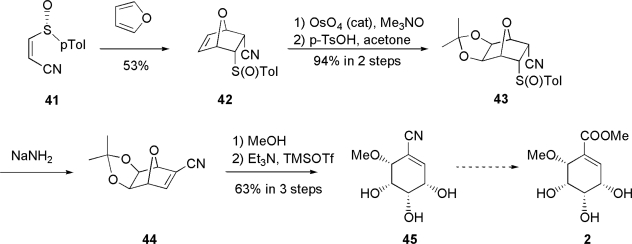
Garcia Ruano’s approach involving asymmetric Diels-Alder cycloaddition.

Garcia Ruano and co-workers reported their attempt to synthesize pericosine B in 2005. As shown in Scheme 4, asymmetric Diels-Alder cycloaddition reaction between chiral 3-sulfinylacrylonitrile **41** and furan with Me_2_AlCl as Lewis acid catalyst gave *endo* adduct **42** in 53% yield together with *exo* adduct in 10% yield [62]. The double bond in **42** was dihydroxylated *exo*-face selectively. The newly generated two hydroxyl groups were protected as acetonide **43**, and this was converted into α,β-unsaturated nitrile **44** by basic treatment with NaNH_2_. Treatment with MeOH, followed by triethylamine and TMSOTf, afforded ring-opened nitrile **45**, which seemed to be the last intermediate in the synthesis of **2**. However, the subsequent transformation of cyano group into methoxycarbonyl group by methanolysis has not been reported so far.

### 3.2. Total synthesis of epimer of pericosine B: Synthesis of pericosine C [63]

**Scheme 5.**
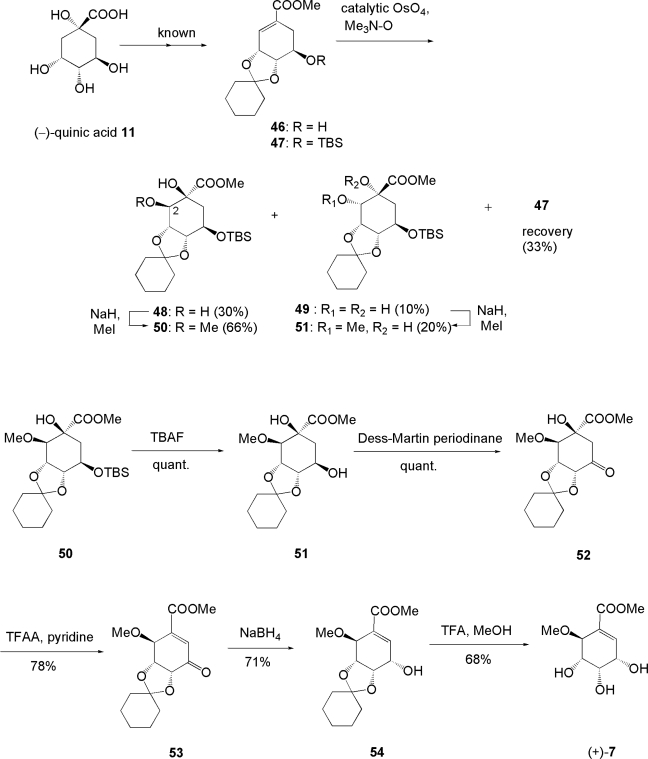
Synthesis of epimer of pericosine B from (−)-quinic acid.

As described above, our synthesis of pericosine B could not be completed. Thus, we attempted to synthesize the epimer of pericosine B **7**, as summarized in Scheme 5. Known methyl shikimate derivative **46** from (−)-quinic acid **11** was converted into TBS ether **47**, which was reacted with catalytic osmium tetroxide and one equivalent of trimethylamine-*N*-oxide under reflux with tBuOH-pyridine-H_2_O (20:5:1) to give a mixture of diols 48 and **49** in 3:1 ratio in 40% yield with recovery of **47** (33%). Major diol **48** was selectively *O*-methylated at C-6 to give **50** in 66% yield as a single product, whereas minor diol **49** was singly 6-*O*-methylated to give **51** in 16% yield with 20% of 1,6-bis-*O*-methylated product. 6-*O*-Methyl ether **51** was further examined for its potential use in the synthesis of pericosine B **2**, but all attempts were unsuccessful. Deprotection of methyl ether **50** with TBAF produced alcohol **51**, and oxidation of **51** with Dess-Martin periodinane gave ketone **52**, which in turn was dehydrated with TFAA to yield unsaturated ketone **53**. Enone **53** was reduced with NaBH_4_ in excellent stereoselectivity to give alcohol **54**, and this was then converted into target molecule (+)-**7**. When tested for *in vitro* for cytotoxicity against P388 cells, obtained (+)-**7** gave an ED_50_ value of 17.8 μg/mL, which indicated lower activity than natural pericosine B (ED_50_: 4 μg/mL). Thus, we concluded that the stereochemistry of C-6 had a significant influence on cytotoxicity. When this work was published in 2004, synthesized (+)-**7** was a non-natural product. However, it was elucidated later that (+)-**7** had the same relative chemistry as natural pericosine A **1** and was a component of natural pericosine C, which is an enantiomeric mixture of **3** and **7** [55].

### 3.3. Total synthesis of initially assigned pericosines A and D [64, 65]

**Scheme 6.**
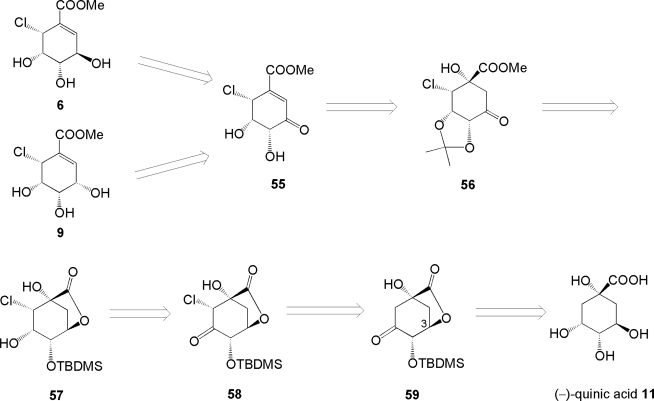
Retro-synthetic strategy of **6** and **9**.

After the publication of the total synthesis of pericosine B **2** by Donohoe, there had been no reports of pericosine A in spite of its antitumor activity. Then, the total synthesis of initially assigned pericosines A **6** and D **9** was attempted by our group because those two target compounds seemed to be synthesized *via* common intermediate enone **55** derived from (−)-quinic acid **11**. Our retro-synthetic strategy is shown in Scheme 6. Key reactions of this synthesis included α-face selective chlorination of silylenolether derived from known ketone **59** [66, 67] and reagent-dependent stereoselective reduction of **55**.

**Scheme 7.**
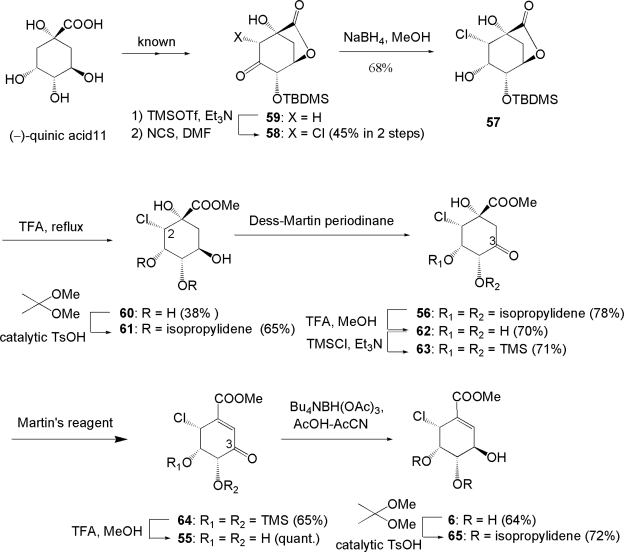
Total synthesis of initially assigned pericosine A **6**.

The synthesis of **6** was carried out as illustrated in Scheme 7. Lactone **59** derived from (−)-quinic acid **11** according to literature [66, 67] was chlorinated with NCS with excellent face selectivity to give α-chloroketone **58** *via* silylenolether in 45% yield in 2 steps. The following reduction of **58** with NaBH_4_ gave alcohol **57** as the sole product. The chemical yield of **57** was improved by sequential reactions from 45% in 2 steps to an overall yield of 57% in 3 steps from ketone **59**. Treatment of **57** with TFA in MeOH under reflux afforded tetraol **60** in 38% yield. Then, **60** was converted into acetonide **61**, which was oxidized with Dess-Martin periodinane to give hydroxyketone **56**. The isopropylidene moiety of **56** was removed with TFA because **56** could not be dehydrated by any dehydrating agents. One plausible explanation was that **56**, conformationally fixed by an isopropylidene bridge, could not be transformed into a suitable transition state for dehydration. Resultant triol **62** was converted into reactive hydroxyketone **63** that was in turn dehydrated to enone **64** with Martin’s sulfrane dehydrating agent (bis[α,α-bis(trifluoromethyl) benzyloxy] diphenyl sulfur) [68] in 65% yield.

Treatment of enone **64** with TFA in MeOH gave **55**, which was reduced stereoselectively with tetrabutylammonium triacetoxyborohydride [69] to afford **6** with the desired configuration as a single product. However, disagreement of the NMR data of **6** and acetonide **65** with those of natural pericosine A and those of its acetonide described in the literature [54] led us to conclude that the proposed stereochemistry of pericosine A was incorrect. Furthermore, the fact that “the isopropylidene bridge in **65** derived from **6** was located between C-4 and C-5, whereas that of the acetonide of pericosine A was between C-3 and C-4” [54] supported our conclusion.

**Scheme 8.**
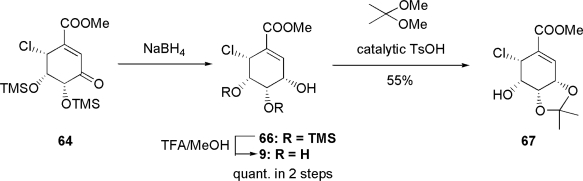
Synthesis of initially assigned pericosine D **9**.

Common intermediate **64** was reduced with NaBH_4_ to afford allyl alcohol **66** as a single product with opposite stereoselectivity. Then, **66** was deprotected to afford **9** quantitatively in 2 steps, as shown in Scheme 8. However, **9** was different from pericosine D. Then, product **9** was transformed into acetonide **67** to confirm the stereochemistry of the synthesized molecule.

The stereochemistry of all intermediates in this study was carefully determined by analyzing various kinds of 2D NMR spectra.

### 3.4. Total synthesis for structure revision and determination of absolute configuration of pericosine A [70, 71]

**Figure 3.**
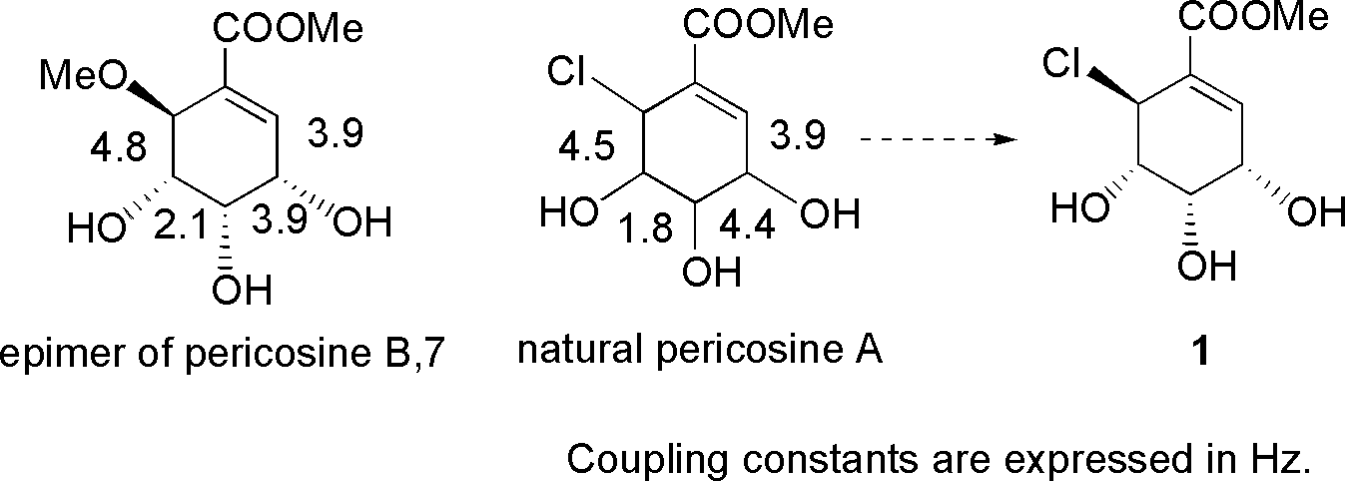
Comparison of coupling constants between epimer of pericosine B **7** and pericosine A **1**.

From our conclusion in the previous study described in Section 3.3, determination of the true structure of pericosine A by total synthesis became our next task. In a comprehensive review of data related to pericosines [54, 63, 64], close similarity between the ^1^H-NMR coupling constants of natural pericosine A [54] and those of **7** [63] led us to deduce that the structure of natural pericosine A was **1**, as illustrated in Figure 3.

**Scheme 9.**
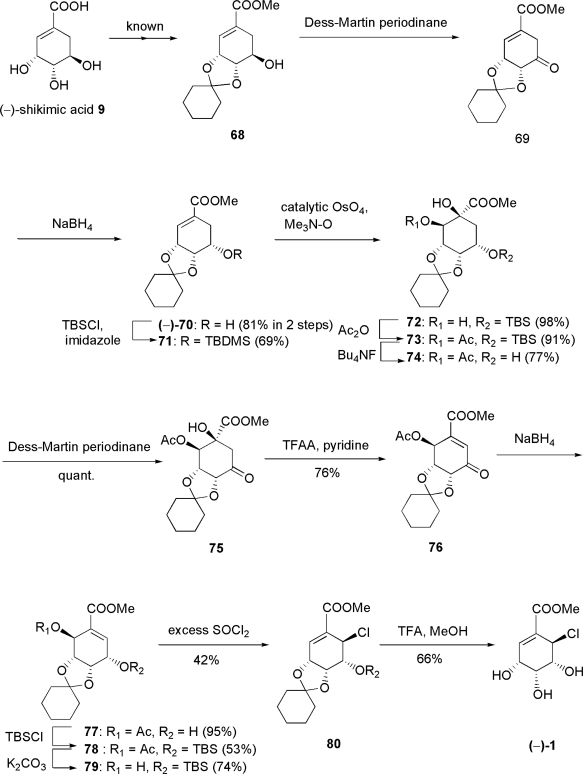
First total synthesis of (−)-pericosine A **1** from (−)-shikimic acid.

Since **1** had the same relative chemistry as **7**, our basic synthetic strategy was almost the same as that for **7**. After a number of trials, the total synthesis of (−)-**1** was achieved, as shown in Scheme 9. Known methyl 5-epishikimate derivative **68** derived from (−)-shikimic acid **9** was subjected to Dess-Martin oxidation to afford β,γ-unsaturated ketone **69**. Without purification, **69** was reduced with NaBH_4_ to give alcohol (−)-**70**, which in turn was protected with TBSCl to give silyl ether **71**. After dihydroxylation of **71**, resultant diol **72** was acetylated to yield **73**, and this was deprotected to give **74**. Subsequent Dess-Martin oxidation of **74** gave β-hydroxyketone **75**, which was dehydrated with TFAA to afford α,β-unsaturated ketone **76**. Subsequent reduction of **76** was carried out carefully with stoichiometric NaBH_4_ at −78°C in dry THF to afford alcohol **77** possessing the desired stereochemistry in 95% yield.

Subsequent protection of **77** was carried out with careful addition of TBSCl and a stoichiometric amount of imidazole to give silyl ether **78** in 53% yield, followed by deacetylation with K_2_CO_3_ to afford enol **79** in 74% yield. The key reaction of this total synthesis, which was aimed at introducing a Cl atom, was achieved by the addition of excess SOCl_2_ to **79** in dry CH_2_Cl_2_ to afford chlorinated product **80** in 42% yield [71], whereas the yield of the reaction with stoichiometric SOCl_2_ was 10% [70]. To our surprise, **80** was formed with rearrangement of the double bond. The structure of key intermediate **80** was confirmed by detailed 1D and 2D NMR studies. In the NOESY spectra, cross peaks H-5/*t*-Bu, H-6/*t*-Bu, H-5/SiMe, H-6/SiMe, and H-3, H4/one of cyclohexyl methylenes, were observed. HMBC cross peak H-3, H-4/singlet carbon of **80**, which was observed at 110.8 ppm, confirmed that the Cl atom was introduced not *via* an S_N_i mechanism as we had aimed early on, but *via* an S_N_2’ mechanism with *syn* selectivity. Another plausible mechanism is the [3,3]-sigmatropic rearrangement of chlorosulfonate derived from **79**. In spite of detailed analysis of NMR spectra, the stereochemistry at C-6 in **80** could not be determined at this step. This total synthesis was completed with TFA to give final product (−)-**1** in 66% yield, which was not **9** as synthesized previously by us [64, 65] but pericosine A. Thus, this result proved the stereochemistry at C-6 in **80**. Except for the sign of the specific rotation, (−)-**1** showed the same spectroscopic data, including HPLC retention time, as the natural product, and the absolute configuration of natural pericosine A was assigned as methyl (3*S*,4*S*,5*S*,6*S*)-6-chloro-3,4,5-trihydroxy-1-cyclohexene-1-carboxylate. The first total synthesis of the antipode of natural pericosine A was completed in this manner.

**Scheme 10.**
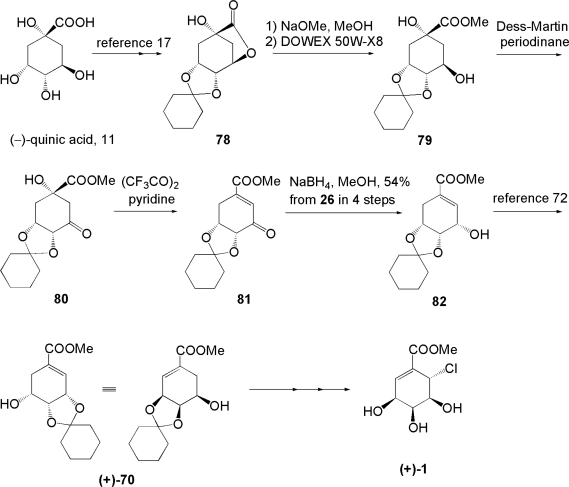
Total synthesis of (+)-pericosine A from (−)-quinic acid.

Next, the total synthesis of natural pericosine A (+)-**1** was examined. As the preparation of (+)-**70**, which is an antipode in the preceding synthesis, from **11** has been reported, a similar strategy toward (−)-**1** could be applicable. The synthesis of (+)-**1** is summarized in Scheme 10 [71]. In our synthesis, the preparation of (+)-**70** was modified from the original method appearing in the literature. Treatment of hydroxylactone **78** derived from **11** with NaOMe in MeOH followed by neutralization with DOWEX® 50W-X8 gave crude diol **79**, which was obtained by only filtration and used in the next reaction without further extraction or purification. Diol **79** was oxidized to β-hydroxyketone **80**, which was then dehydrated with TFAA to produce enone **81**. Reduction with NaBH_4_ afforded enol **82** in 54% overall yield from **78**. Enol **82** was converted into (+)-**70** according to literature [72]. The following transformation of (+)-**70** into (+)-**1** was accomplished as above. Since all spectral data of synthesized (+)-**1**, including specific rotation and HPLC retention time, agreed with the data of natural pericosine A, the first synthesis of natural pericosine A was completed.

## 4. Further Discussion

As described above, pericosines are interesting compounds because of their potent biological activities and unique structures. In particular, the role of pericosine A **1** in anti-tumor, protein kinase EGFR inhibition or topoisomerase II inhibition was demonstrated. In terms of chemical structure, the relative chemistry of pericosines A **1**, C **3**, and B **2** is so unique that it is difficult to find the same configurational carbasugar natural products, whereas pericosine D **4** has the same relative configuration as (+)-MK7607. We are extremely interested in the fact that pericosines C **3** and E **5** exist as an enantiomeric mixture. How are they biologically formed? Pericosine E **5** is thought to be a conjugate of pericosines A **1** and B **2** but with different chiral sense. This suggests the possibility of the presence of antipodes of **1** and **2** or unknown analogues with other combinations. We are currently directing our efforts toward designing a more effective synthetic route for pericosine A **1** because of the low total yield in our completed first synthesis. Nevertheless, the SN2’ type reaction, which is the key step for Cl introduction in our previous work [70, 71], may help unravel the mystery surrounding the chirality of percosines. A reaction similar to this or the enantioselective dehydration from prochiral 3-epiquinate may occur in fungal metabolic systems to generate chirality. Indeed, it seems unbelievable that enantiomeric mixtures of **3** and **5**, particularly **5** that bears 8 chiral centers, could be formed biologically. In that sense, pericosines are truly exciting compounds.

**Figure 4.**
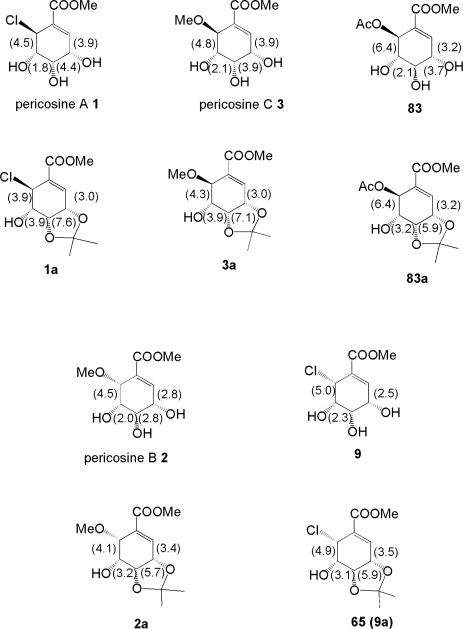
Coupling constants in Hz observed in ^1^H-NMR spectra of pericosines and their analogues.

On the other hand, we would like to point out the ambiguity of the conformation of pericosines. It is absolutely difficult to determine the configuration or conformation of pericosines only from spectral analysis. ^1^H-NMR coupling constants of pericosines and acetonides are shown in Figure 4. The correct structure of **1** could not be concluded only from the synthesis of **1**. The stereochemistry of C-6 in intermediate **80** could be determined not from detailed spectral analysis but from the difference between deprotected **1** and **9**, the latter of which was synthesized in our previous study [64, 65]. We hope to solve this problem by integrating our vast synthetic efforts with data from other researchers.

The synthetic study of pericosine D is ongoing in an effort to determine its absolute configuration [73]. After elucidating all the structures of pericosines, we will examine the biological activity of synthesized pericosine analogues, develop more effective synthetic routes, and design more active molecules based on these seed compounds.

## Figures and Tables

**Table 1.** Cytotoxicity of pericosines against murine P388 cell line.

	Pericosine A	Pericosine B	Pericosine C	Pericosine D	Pericosine E
ED_50_ (μg/mL)	0.1	4.0	10.5	3.0	15.5

## References

[1] Suami T (1987). Synthetic ventures in pseudo-sugar chemistry. Pure Appl Chem.

[2] Berecibar A, Grandjean C, Sinwardena A (1999). Synthesis and Biological Activity of Natural Aminocyclopentitol Glycosidase Inhibitors: Mannostatins, Trehazolin, Allosamidins, and Their Analogs. Chem Rev.

[3] Arjona O, Gomez AM, Lopez JC, Plumet J (2007). Synthesis and conformational and biological aspects of carbasugars. Chem Rev.

[4] Mirza S, Molleyres LP, Vasella A (1985). Synthesis of a glyoxalase I inhibitor from Streptomyces griseosporeus Niida et Ogasawara. Helvetica Chimica Acta.

[5] Takayama H, Hayashi K, Koizumi T (1986). Enantioselective total synthesis of glyoxalase I inhibitor using asymmetric Diels-Alder reaction of a new chiral dienophile, (S)S-3-(3-trifluoromethylpyrid-2-ylsulfinyl)acrylate. Tetrahedron Lett.

[6] Yamakoshi Y, Nakajima Y, Ge W-Y, Sugita J, Okayama K, Takahashi T, Koizumi T (1996). High pressure mediated asymmetric Diels-Alder reaction of chiral sulfinylacrylate derivatives with furan and 2-methoxyfuran. Heterocycles.

[7] Takahashi T, Yamakoshi Y, Okayama K, Yamada J, Ge W-Y, Koizumi T (2002). High-pressure mediated asymmetric Diels-Alder reaction of chiral sulfinylacrylate derivatives and its application to chiral synthesis of (−)-COTC and (−)-gabosine C. Heterocycles.

[8] Arthurs CL, Wind NS, Whitehead RC, Stratford IJ (2007). Analogues of 2-crotonyloxymethyl-(4R,5R,6R)-4,5,6-trihydroxycyclohex-2-enone (COTC) with anti-tumor properties. Bioorg & Med Chem Lett.

[9] Arthurs CL, Raftery J, Whitby HL, Whitehead RC, Wind NS, Stratford IJ (2007). Arene cis-dihydrodiols: Useful precursors for the preparation of analogs of the antitumor agent, 2-crotonyloxymethyl-(4R,5R,6R)-4,5,6-trihydroxycyclohex-2-enone (COTC). Bioorg & Med Chem Lett.

[10] Ramanaa GV, Rao BV (2005). Stereoselective synthesis of (−)-gabosine C using a Nozaki-Hiyama-Kishi reaction and RCM. Tetrahedron Lett.

[11] Huntley CFM, Wood HB, Ganem B (2000). A new synthesis of the glyoxalase-I inhibitor COTC. Tetrahedron Lett.

[12] Huntley CF, Hamilton DS, Creighton DJ, Ganem B (2000). Reaction of COTC with glutathione: structure of the putative glyoxalase I inhibitor. Org Lett.

[13] Shing T, Tang Y (1990). Enantiospecific synthesis of 2-crotonyloxy-(4R,5R,6R)-4,5,6-trihydroxycyclohex-2-enone (COTC) from quinic acid. J Chem Soc, Chem Commun.

[14] Shing T, Tang Y (1990). (−)-Quinic acid in organic synthesis. 1. A facile synthesis of 2-crotonyloxymethyl-(4R,5R,6R)-4,5,6-trihydroxycyclohex-2-enone. Tetrahedron.

[15] Tatsuta K, Yasuda S, Araki N, Takahashi M, Kamiya Y (1998). Total synthesis of a glyoxalase I inhibitor and its precursor, (−)-KD16-U1. Tetrahedron Lett.

[16] Shinada T, Fuji T, Ohtani Y, Yoshida Y, Ohfune Y (2002). Syntheses of gabosine A, B, D, and E from allyl sulfide derived from (−)-quinic acid. Synlett.

[17] Lygo B, Swiatyj M, Trabsa H, Voyle M (1994). Synthesis of (+)-Gabosines C and E from D-ribose Tetrahedron Lett.

[18] Banwell MG, Bray AM, Wong D (2001). A concise and chemo-enzymatic synthesis of (−)-gabosine A, a carba-sugar enone from Streptomycetes. J New J Chem.

[19] Lubineau A, Billault I (1998). New Access to Unsaturated Keto Carba Sugars (Gabosines) Using an Intramolecular Nozaki-Kishi Reaction as the Key Step. J Org Chem.

[20] Alibes R, Bayon P, De March P, Figueredo M, Font J, Marjanet G (2006). Enantioselective synthesis and absolute configuration assignment of gabosine O. Synthesis of (+)- and (−)-gabosine N and (+)- and (−)-epigabosines N and O. Organic Lett.

[21] Shing TKM, Cheng HM (2007). Short Syntheses of Gabosine I and Gabosine G from δ-D-Gluconolactone. J Org Chem.

[22] Mehta G, Lakshminath S (2000). A norbornyl route to cyclohexitols: stereoselective synthesis of conduritol-E, allo-inositol, MK 7607 and gabosines. Tetrahedron Lett.

[23] Song C, Jiang S, Singh G (2001). Synthesis of (−)-MK7607 and Other Carbasugars from (−)-shikimic Acid. Synlett.

[24] Schmidt RR, Koen A (1987). α-Glucosidase inhibitors. Part 4. Synthesis of valienamine. Angew Chem.

[25] Park TK, Danishefsky SJ (1994). A synthetic route to valienamine: an interesting observation concerning stereoelectronic preferences in the SN2’ reaction. Tetrahedron Lett.

[26] Fukase H, Horii S (1992). Synthesis of a branched-chain inosose derivative, a versatile synthon of N-substituted valiolamine derivatives from D-glucose. J Org Chem.

[27] Trost BM, Chupak S, Luebbers T (1998). Total Synthesis of (±)- and (+)-Valienamine via a Strategy Derived from New Palladium-Catalyzed Reactions. J Am Chem Soc.

[28] Shing TKM, Li TY, Kok SH-L (1999). Enantiospecific Syntheses of Valienamine and 2-epi-Valienamine. J Org Chem.

[29] Yoshikawa M, Cha BC, Okaichi Y, Takinami Y, Yokokawa Y, Kitagawa I (1988). Syntheses of validamine, epi-validamine, and valienamine, three optically active pseudo-amino-sugars, from D-glucose. Chem Pharm Bull.

[30] Tatsuta K, Mukai H, Takahashi M (2000). Novel synthesis of natural pseudo-aminosugars, (+)-valienamine and (+)-validamine. J Antibiot.

[31] Chang Y-K, Lee B-Y, Lee GS, Jeon HB, Kim KS (2005). An Efficient Synthesis of Valienamine via Ring-Closing Metathesis. J Org Chem.

[32] von Itzstein M, Wu WY, Kok GB, Pegg MS, Dyason JC, Jin B, Van PT, Smythe ML, White HF, Oliver SW, Colman PM, Varghese JN, Ryan DM, Woods JM, Bethell RC, Hotham VJ, Cameron JM, Penn C (1993). R Rational design of potent silalidase-based inhibitors of influenza virus replication. Nature.

[33] Kim CU, Lew W, Williams MA, Zhang L, Liu H, Swaminathan S, Bischofberger N, Chen MS, Tai CY, Mendel DB, Laver WG, Stevens RC (1997). Influenza neuraminidase inhibitors possessing a novel hydrophobic interaction in the enzyme active site: design, synthesis, and structural analysis of carbocyclic sialic acid analogues with potent anti-influenza activity. J Am Chem Soc.

[34] Rohloff JC, Kent KM, Postich MJ, Becker MW, Chapman HH, Kelly DE, Lew W, Louie MS, McGee LR, Prisbe EJ, Schultze LM, Yu RH, Zhang L (1998). Practical Total Synthesis of the Anti-Influenza Drug GS-4104. J Org Chem.

[35] Yeung Y-Y, Hong S, Corey EJ (2006). A Short Enantioselective Pathway for the Synthesis of the Anti-Influenza Neuramidase Inhibitor Oseltamivir from 1,3-Butadiene and Acrylic Acid. J Am Chem.Soc.

[36] Fukuta Y, Mita T, Fukuda N, Kanai M, Shibasaki M (2006). Catalytic Asymmetric Total Synthesis of (+)-Lactacystin. J Am Chem Soc.

[37] Mita T, Fukuda N, Roca FX, Kanai M, Shibasaki M (2007). Second generation catalytic asymmetric synthesis of Tamiflu: allylic substitution route. Org Lett.

[38] Satoh N, Akiba T, Yokoshima S, Fukuyama T (2007). A practical synthesis of (−)-oseltamivir. Angewandte Chem, Int Ed.

[39] Karchier M, Michalak K, Wicha J (2007). Anti-influenza drugs Synthesis of Tamiflu, a drug kept in stock to prevent a bird flu epidemic. Wiadomosci Chemiczne.

[40] Shie J-J, Fang J-M, Wang S-Y, Tsai K-C, Cheng Y-SE, Yang A-S, Hsiao S-C, Su C-Y, Wong C-H (2007). Synthesis of Tamiflu and its Phosphonate Congeners Possessing Potent Anti-Influenza Activity. J Am Chem Soc.

[41] Takeuchi T, Chimura H, Hamada M, Umezawa H, Yoshioka O, Oguchi N, Takahashi Y, Matsuda A (1975). Glyoxalase I inhibitor of a new structural type produced by Streptomyces. J Antibiot.

[42] Tatsuta K, Tsuchiya N, Mikami N, Umezawa S, Umezawa H, Naganawa H (1974). KD16-U1, a new metabolite of Streptomyces. Isolation and structural studies. J Antibiot.

[43] Bach G, Breiding-Mack S, Grabley S, Hammann P, Hutter K, Thiericke R, Uhr H, Wink J, Zeeck A (1993). Secondary metabolites by chemical screening. 22. Gabosines, new carba-sugars from Streptomyces. Liebigs Ann Chem.

[44] Yoshikawa N, Chiba N, Mikawa T, Ueno S, Harimaya K, Iwata M (1994). Novel herbicidal MK7607 and its manufacture with Curvularia. Jpn Kokai Tokkyo Koho JP.

[45] Horii S, Iwasa T, Mizuta E, Kameda Y (1971). Validamycins, new antibiotics. VI. Validamine, hydroxyvalidamine, and validatol, new cyclitols. J Antibiot.

[46] Kameda Y, Horii S (1972). Structure of the antibiotic validamycin A. J Chem Soc, Chem Commun.

[47] Suami T, Ogawa S, Chida N (1980). The revised Structure of Validamycin A. J Antibiot.

[48] Laube H, Fouladfar M, Aubell R, Schmitz H (1980). Effect of glucosidase inhibitor, Bay g 5421 (acarbose), on the blood glucose in obese diabetic patients type 2 (NIDDM). Arzneimittel-Forschung.

[49] Moore RE, Corbett TH, Patterson GML, Valeriote FA (1996). The search for new antitumor drugs from blue-green algae. Current Pharmaceutical Design.

[50] Newman DJ, Cragg GM (2007). Natural Products as Sources of New Drugs over the Last 25 Years. J Nat Prod.

[51] Fenical W, Jensen PR (2006). Developing a new resource for drug discovery: marine actinomycete bactereia. Nature Chemical Biology.

[52] Newman DJ, Cragg GM (2006). Natural products from marine invertebrates and microbes as modulators of antitumor targets. Current Drug Targets.

[53] Newman DJ, Hill RT (2006). New drugs from marine microbes: the tide is turning. J Ind Microbiol Biotechnol.

[54] Numata A, Iritani M, Yamada T, Minoura K, Matsumura E, Yamori T, Tsuruo T (1997). Novel antitumor metabolites produced by a fungal strain from a sea hare. Tetrahedron Lett.

[55] Yamada T, Iritani M, Ohishi H, Tanaka K, Doi M, Minoura K, Numata A (2007). Pericosines, antitumor metabolites from the sea hare-derived fungus Periconia byssoides. Structures and biological activities. Org Bioorg Chem.

[56] Yamada T, Minoura K, Numata A

[57] Yamada T (2002).

[58] Donohoe TJ, Blades K, Helliwell M, Waring MJ, Newcombe NJ (1998). The Total Synthesis of (+)-Pericosine B. Tetrahedron Lett.

[59] Okamura H, Nakamura Y, Morishige K, Ohura R, Shimizu H, Iwakawa T, Nakatani M (1988). Development of Base Catalized Diels-Alder Reaction of 3-Hydroxy-2-pyrone and its Application to Synthesis of Biologically Active Compounds.

[60] Usami Y, Numata A (2004). Examination of the reactivities of hydroxy groups in multioxygenated cyclohexanoids: Synthetic study toward cytotoxic pericosine B. Chem Pharm Bull.

[61] Garcia Ruano J, Alemparte C, Lopez-Cantarero J (2000). (Z)-3-p-Tolylsulfinylacrylonitrile as a Chiral Dienophile: Diels-Alder Reactions with Furan and Acyclic Dienes. J Org Chem.

[62] Garcia Ruano J, Lopez-Cantarero J, Martin Castro AM, Adams H, Rogriguez Ramos JH (2005). toward the Synthesis of (+)-Pericosine B. Phosphorus, Sulfur and Silicon and the Related Elements.

[63] Usami Y, Hatsuno C, Yamamoto H, Tanabe M, Numata A (2004). Synthesis of the epimer of pericosine B from (−)-quinic acid. Chem Pharm Bull.

[64] Usami Y, Ueda Y (2005). Synthetic Study toward Antitumour Natural Product Pericosine A. Chem Lett.

[65] Usami Y, Ueda Y (2007). Stereoselective Syntheses of Diastereomers of Antitumor Natural Product Pericosine A from (−)-Quinic Anid. Synthesis.

[66] Manthey MK, Gonzalez-Bello C, Abell C (1997). Synthesis of (2R)-2bromodehydroquinic acid and (2R)-2fluorodehydroquinic acid. J Chem Soc, Perkin Trans.

[67] Gonzalez-Bello C, Manthey MK, Harris JH, Hawkins AR, Coggins JR, Abell C (1998). Synthesis of 2-Bromo- and 2-Fluoro-3-dehydroshikimic Acids and 2-Bromo- and 2-Fluoroshikimic Acids Using Synthetic and Enzymic Approaches. J Org Chem.

[68] Alhalt RJ, Martin JC (1972). Sulfuranes. VI. Reactions involving the alkoxy ligands of dialkoxydiarylsulfuranes. Formation of olefins and ethers. J Am Chem Soc.

[69] Evans DA, Chapman KT (1986). The directed reduction of β-hydroxy ketones employing Me_4_NHB(OAc)_3_. Tetrahedron Lett.

[70] Usami Y, Horibe Y, Takaoka I, Ichikawa H, Arimoto M (2006). First Total Synthesis of (−)-Pericosine A from (−)-Shikimic Acid: Structure Revision and Determination of the Absolute Configuration of Antitumor Natural Product Pericosine A. Synlett.

[71] Usami Y, Takaoka I, Ichikawa H, Horibe Y, Tomiyama S, Ohtsuka M, Imanishi Y, Arimoto M (2007). First Total Synthesis of (+)- and (−)-Pericosine A: Determination of Absolute Stereo Structure. J Org Chem.

[72] Ulibarri G, Nadler W, Skrydstrup T, Audrain H, Chianori A, Riche C, Grierson AA (1995). Construction of the Bicyclic Core Structure of the Enediyne Antibiotic Esperamicin-A_1_ in Either Enantiomeric Form from (−)-Quinic Acid. J Org Chem.

[73] Usami Y, Mizuki K, Ichikawa H, Arimoto M

